# A community intervention trial of multimodal suicide prevention program in Japan: A Novel multimodal Community Intervention program to prevent suicide and suicide attempt in Japan, NOCOMIT-J

**DOI:** 10.1186/1471-2458-8-315

**Published:** 2008-09-15

**Authors:** Yutaka Ono, Shuichi Awata, Hideharu Iida, Yasushi Ishida, Naoki Ishizuka, Hiroto Iwasa, Yuichi Kamei, Yutaka Motohashi, Atsuo Nakagawa, Jun Nakamura, Nobuyuki Nishi, Kotaro Otsuka, Hirofumi Oyama, Akio Sakai, Hironori Sakai, Yuriko Suzuki, Miyuki Tajima, Eriko Tanaka, Hidenori Uda, Naohiro Yonemoto, Toshihiko Yotsumoto, Naoki Watanabe

**Affiliations:** 1 Health Center, Keio University,.35 Shinanomachi, Shinjuku-ku, Tokyo 160-8582, Japan; 2 Division of Neuropsychiatry and Center for Dementia, Sendai City Hospital, 3-1 Shimizukouji, Wakabayashi-ku, Sendai 984-8501, Japan; 3 Department of Occupational Therapy, Faculty of Nursing and Rehabilitation, Aino University, 4-5-4 Higashiooda, Ibaraki-shi, Osaka 567-0012, Japan; 4 Department of Psychiatry, Faculty of Medicine, University of Miyazaki, 5200 Kihara, Kiyotake-cho, Miyazaki-gun, Miyazaki 889-1692, Japan; 5 Division of Preventive Medicine, Department of Community Health and Medicine, esearch Institute, International Medical Center of Japan, 1-21-1 Toyama, Shinjuku-ku, Tokyo 162-8655, Japan; 6 Aomori Prefectural Center for Mental Health and Welfare, 353-92 Sawabe Sannai, Aomori-shi, Aomori 038-0031, Japan; 7 Department of Psychiatry, Kohnodai Hospital, National Center of Neurology and Psychiatry, 1-7-1 Kohnodai, Ichikawa-shi, Chiba 272-8516, Japan; 8 Department of Public Health, Akita University School of Medicine, 1-1-1 Hondo, Akita-shi, Akita 010-8543, Japan; 9 Department of Psychiatry, Graduate School of Medicine, Keio University, 35 Shinanomachi, Shinjuku-ku, Tokyo 160-8582, Japan; 10 Department of Psychiatry, University of Occupational and Environmental Health, 1-1 Iseigaoka, Yahatanishi-ku, Kitakyusyu-shi, Fukuoka 807-8555, Japan; 11 Health Promotion Division of Health and Social Welfare Department, Kagoshima Prefecture, 10-1 Kamoike-shinmachi, Kagoshima-shi, Kagoshima 890-8577, Japan; 12 Department of Neuropsychiatry, Iwate Medical University, 19-1 Uchimaru, Morioka-shi, Iwate 020-8505, Japan; 13 Department of Social Welfare, Faculty of Health Sciences, Aomori University of Health and Welfare, 58-1 Mase Hamadate, Aomori-shi, Aomori 030-8505, Japan; 14 Graduate School of Medicine, Gunma University, 39-22 Showa-machi 3-chome, Maebashi-shi, Gunma 371-8511, Japan; 15 Department of Adult Mental Health, National Institute of Mental Health, National Center of Neurology and Psychiatry, 4-1-1 Ogawahigashi-cho, Kodaira-shi, Tokyo 187-8553, Japan; 16 Stress Management Office, Keio University, 35 Shinanomachi, Shinjuku-ku, Tokyo 160-8582, Japan; 17 Kanoya Public Health Center, 2-16-6 Utsuma, Kanoya-shi, Kagoshima 893-0011, Japan; 18 Department of Biostatistics, School of Public Health, Kyoto University, Yoshidakonoecho, Sakyo-ku, Kyoto-shi, Kyoto 606-8501, Japan; 19 Sensatsu Public Health Center, 228-1 Kumanojocho, Satsumasendai-shi, Kagoshima 895-0041, Japan; 20 Department of Psychology, Faculty of Human Sciences, Kansai University of International Studies, 1-18 Aoyama, Shizimi-cho, Miki-shi, Hyogo 673-0521, Japan

## Abstract

**Background:**

To respond to the rapid surge in the incidence of suicide in Japan, which appears to be an ongoing trend, the Japanese Multimodal Intervention Trials for Suicide Prevention (J-MISP) have launched a multimodal community-based suicide prevention program, NOCOMIT-J. The primary aim of this study is to examine whether NOCOMIT-J is effective in reducing suicidal behavior in the community.

Methods/DesignThis study is a community intervention trial involving seven intervention regions with accompanying control regions, all with populations of statistically sufficient size. The program focuses on building social support networks in the public health system for suicide prevention and mental health promotion, intending to reinforce human relationships in the community. The intervention program components includes a primary prevention measures of awareness campaign for the public and key personnel, secondary prevention measures for screening of, and assisting, high-risk individuals, after-care for individuals bereaved by suicide, and other measures. The intervention started in July 2006, and will continue for 3.5 years. Participants are Japanese and foreign residents living in the intervention and control regions (a total of population of 2,120,000 individuals).

**Discussion:**

The present study is designed to evaluate the effectiveness of the community-based suicide prevention program in the seven participating areas.

**Trial registration:**

UMIN Clinical Trials Registry (UMIN-CTR) UMIN000000460.

## Background

### Recent rapid increase of suicide in Japan

#### (1) Changes in suicide incidence

According to vital statistics collected by the Japan Ministry of Health, Labour, and Welfare in 1997, there were 23,494 suicides (15,901 men and 7,593 women), with the number rising to 31,755 (22,349 men and 9,406 women) in 1998, which represented a 35% increase. This was the highest rate of increase recorded since the Ministry began tracking mortality statistics. The number of suicides remained high in subsequent years, reaching 29,949 in 2002 and 32,109 in 2003.

In 2002, the World Health Organization (WHO) reported that the suicide rate in Japan (25.3 per 100,000) was higher than in any other developed nation (for comparison: France: 17.5, Germany: 13.5, Canada: 11.7, United States of America: 10.4, United Kingdom: 7.5, Italy: 7.1).

In terms of the number of suicides, three peaks have emerged since World War II. However, the most recent rise that started in 1998 has shown no signs of abating, and represents the worst in Japan's history. Therefore, it is clear that suicide prevention measures are urgently needed in Japan.

#### (2) Regional tendencies

It has been pointed out that the suicide rate has traditionally been high in the three prefectures of the northern Tohoku area (Akita, Iwate, and Aomori), Niigata, Shimane, and the Kyushu area (Miyazaki, Kagoshima, and Okinawa) [1].

The increase in the number of suicides that began in 1998, however, was not necessarily attributable to suicides in these rural areas. Fujita (2003) [2] conducted a comparative study of suicide rates by prefecture by comparing a time period with a low number of suicides (1989–1995) to time periods before and after, during which the number of suicides was on the rise (1983–1987 and 1998–2000, respectively). The findings indicated that the recent increase in the number of suicides has been significantly more prominent in urban areas such as Tokyo, Osaka, and their surrounding areas, than in rural areas. During the two periods 1989–1995 and 1998–2000, the mean number of suicides among people 15 years of age or older rose from 894 to 1,658 in Osaka, from 713 to 1,309 in Kanagawa, and from 1,129 to 1,938 in Tokyo.

With regard to recent trends in suicide rate by age, the middle-aged population was found to have higher suicide rates. In 2004, 42.1% of those who committed suicide were 45 to 64 years old. This tendency was particularly evident among men, in whom the suicide rate peaked at 55 to 59 years of age, whereas a similar trend was not found in women, in whom the suicide rate generally increased with age.

#### (3) Causes and motives for suicide

According to the statistics of the National Police Agency, health and financial/lifestyle problems were the top two reasons for suicide. Although this tendency remained the same during the increase in suicides that began in 1998, the number of suicides due to financial/lifestyle problems has increased more rapidly compared to suicides committed due to health problems. Among those who committed suicide with or without suicide notes in 1997, 13,659 individuals (56.0%) did so due to health problems and 3,556 individuals (14.6%) due to financial/lifestyle problems. These numbers rose to 16,769 (51.0%, a 22.8% increase over the previous year) and 6,058 (18.4%, a 70.4% increase over the previous year), respectively, in 1998. In terms of those with health problems, the number of suicides subsequently decreased in 2004 to 14,786 (45.7%), whereas the number of suicides due to financial/lifestyle problems increased to 7,947 (24.6%). This indicates that the percentage of suicides due to financial/lifestyle problems has been increasing.

### Recent suicide prevention programs in Japan

Many suicide prevention measures have been implemented internationally [3-5]. In Japan, evidence has also emerged recently to support the effectiveness of community-based programs for suicide prevention. Seven community-based intervention trials implemented for five years or more have been conducted between 1985 and 2005 in Japan. All the trials used a quasi-experimental design and included suicide rate as the primary outcome. These suicide prevention programs included the development of social support networks in the community and/or depression screening for residents with follow-up by physicians. All the intervention programs were also administered by local governments. Six of the seven trials targeted individuals aged 65 years and older.

The first trial was conducted in Matsunoyama, Niigata prefecture. During the 10-year implementation period, the suicide rate of over 150 per 100,000 decreased by 75% for both men and women aged 65 years and older [6]. In the trials conducted in Joboji (Iwate pref.), Nagawa (Aomori pref.), Matsudai and Yasuzuka (Niigata pref.), and Yuri (Akita pref.), the suicide prevention program significantly reduced the suicide risk for individuals aged 65 years and older [7-10].

Recently, a relatively large, multimodal intervention trial targeting all age groups was conducted in four municipalities of Akita. During the four-year implementation period, the suicide rate of 68 per 100,000 for all residents was reduced by 27% [11].

The results of these seven trials suggest that community-based intervention would be effective for preventing suicide and that the increase of suicide deaths in Japan may be related to more pervasive social isolation than in the past, and to an absence of personal psycho-social development compared with financial success.

However, the sample sizes in these trials were relatively small and the monitoring of the implementation process was insufficient. Furthermore, since the trials were conducted in rural areas with high suicide rates, it is still unclear whether similar community-based programs would be effective in urban areas where the suicide rates have increased rapidly. Therefore large, community-based intervention trials with adequate controls should be conducted to develop an effective, evidence-based suicide prevention program to reduce the future suicide rate in Japan.

### Objectives of this study

(1) The primary goal is to examine the effectiveness of a community-based multimodal intervention program for suicide prevention in regions where the suicide rate was relatively high compared to control regions. These target areas were designated "Group 1".

(2) The secondary goal was to explore the effectiveness of a community-based multimodal intervention program for suicide prevention in highly populated regions. These target areas were designated "Group 2".

## Methods/Design

A community intervention trial will be conducted to evaluate the effectiveness of a novel suicide intervention program. In this study, the incidence of suicidal behavior in an intervention group and a control group will be compared.

### Organization

The Japan Ministry of Health, Labour, and Welfare selected the Japan Foundation for Neuroscience and Mental Health (JFNMH) as the primary institution responsible for the strategic research program for suicide prevention. The JFNMH conducts the program "Japanese Multimodal Intervention Trials for Suicide Prevention, J-MISP" in close collaboration with the National Center of Neurology and Psychiatry. NOCOMIT-J is one of two research projects being conducted by J-MISP. The other is a randomized, controlled, multicenter trial of post-suicide attempt case management for prevention of further attempts in Japan (ACTION-J).

The principal investigator of NOCOMIT-J and the sub-leader will supervise the study group in order to conduct and complete the study effectively.

The study group management office will engage in overall administrative procedures regarding the operation of the study group. It will also set up and operate the study group steering committee and the intervention program committees, hold the research conference, and respond to questions from institutions in the participating regions.

The J-MISP director, the principal investigator of the NOCOMIT-J, and the regional leaders share the information and collaborate to resolve problems and safety issues with the help of the steering committee and the Central and Local Research Ethics Committee.

The study group steering committee will be composed of regional leaders and other key members of the study group. Research meetings will be held upon the principal investigator's request. At the meetings, the intervention program committee will present the agenda, after which important issues, such as revision of the protocol or stopping of the study, will be discussed.

### Participants and Participating Areas

#### Participants

The participants will include Japanese and foreign residents living in the intervention and control regions.

#### Eligibility Criteria

Target areas will be selected and divided into two groups: "Group 1" and "Group 2" as mentioned above. The areas meeting the following criteria will be eligible for the study:

a) Areas with strong support from local government and other organizations to conduct this multimodal suicide prevention program.

b) Areas capable of selecting intervention and control regions.

c) Areas capable of following the data collection procedure.

d) Areas with comparable baseline data on suicide attempts in intervention and control regions.

e) Areas with comparable baseline demographic data in intervention and control regions.

#### Participating regions and sample size estimation

(1) Group 1: Regions in Aomori, Akita, Iwate, and the Minamikyushu area, with a total population of 670,000 individuals.

(2) Group 2: Regions in Sendai, Chiba, and the Kitakyushu area, with a total population of 1,450,000 individuals.

**Figure 1:**
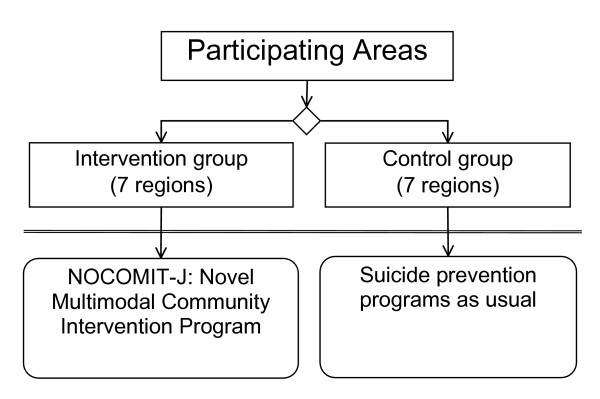
Flow diagram of the study.

After a preliminary survey to record the rate of suicidal behavior and other information in the target regions in these areas, intervention and control regions will be selected based on the similarity in community characteristics and the incidence of suicidal behavior (Figure 1).

Each region has a designated regional leader. The personnel associated with each region include psychiatrists, researchers supporting community intervention, and personnel in charge of regional health administration.

#### Rationale for estimation of sample sizes

Sample sizes to be used in the study were calculated based on the assumptions of the outcome and suicide rates from 2002 to 2004 in Groups 1 and 2 presented in Table 1.

**Table 1:** Assumptions of the outcome and suicide rates in Group 1 and Group 2

	Group 1	Group 2
Suicide rate in control regions (per 100,000 individuals)	30	20
Proportion of expected numbers of ambulance transports due to "self-harm" (severe and mild cases) relative to completed suicides	50%	50%
Expected suicide rate reduction over observed 3 years by intervention	20%	15%
Significance level (two-sided)	5%	5%

*Notes*. These assumptions of the outcome and suicide rates (2002–2004) are used to calculate the sample sizes in this study.

Group1: regions with a relatively high suicide rate compared to control regions, examined to gauge the effectiveness of the community-based multimodal intervention program for suicide prevention.

Group 2: highly-populated regions, examined in order to explore the effectiveness of the community-based multimodal intervention program for suicide prevention.

Although the estimated sample sizes are not adjusted for 5-year age group, sex, and regional characteristics, if all assumptions are met, the statistical power will be over 80% for each group, regarding person-year incidences in the intervention and control regions.

Using the O'Brien-Fleming method [12] in the interim analysis, the significance level in the final analysis is estimated to be 4.9% for a two-sided test. In addition, the statistical power will be over 80% in each group.

### Intervention

The intervention program will be implemented by local authorities.

#### Suicide prevention program in control regions

The interventions in the control regions include the usual suicide prevention programs.

#### Suicide prevention programs in intervention regions

The local health authorities will implement the suicide prevention measures in accordance with the intervention manual developed by the program committee of the study group. To better enhance the quality of the essential intervention activities, the local health authority is also requested to share with the other study group members the information on the program tools.

##### The program components

The program stresses that bonds between human beings, social support, and social capital within communities are key factors for reducing suicide. Its essential components are listed below.

(1) The program focuses on building social support networks in the public health system for suicide prevention and mental health promotion, which will reinforce human relationships in the community.

- Network meetings of related departments and organizations will be held.

- Coordinating committees for the program will be formed in the intervention regions.

(2) Primary prevention measures for suicide and suicide-related behavior

- An public awareness campaign will be set up.

- Community programs will be set up to allow residents to gather and communicate.

(3) Secondary prevention measures for suicide and suicide-related behavior

- High-risk individuals will be screened.

- Counseling and outreach services will be provided.

(4) After-care for individuals bereaved by suicide

(5) Suicide prevention measures targeting individuals with substance/alcohol-related disorders, schizophrenia, and other mental health disorders

(6) Suicide prevention measures targeting individuals with work-related problems.

### Study period

Study period: August 2005 to March 2010.

Intervention period: July 2006 to December 2009.

### Approval of the study protocol

This study protocol was reviewed and approved by the Central Research Ethics Committee of the J-MISP. Additionally, the regional leaders will ask the local governors for cooperation, and obtain written authorization to conduct the study. Regional leaders will obtain approval from the ethics committees of affiliated universities or hospitals.

### Data collection

#### Baseline Information

Data will be collected for the items below:

##### (1) Statistics on suicide

The number of suicides in the 3 years prior to the study (2003–2005) in the study regions was recorded by sex and 5-year age group the Japan Ministry of Health, Labour, and Welfare.

##### (2) Information from the emergency report

Information on "self-harmed" individuals transported by ambulance in the 3 years prior to the study was collected from the emergency reports of ambulance service.

##### (3) Demographic information

A total population count in the regions in the 3 years prior to the study was recorded by sex and 5-year age group by each local governments.

##### (4) Regional characteristics

The following information was collected from published statistical data sources: geographic information, proportion of unmarried individuals, widowed spouses, divorcees, nuclear families, the unemployed, individuals in the labor force, and the annual population turnover in the regions.

##### (5) Suicide prevention programs in existence prior to the study

Baseline information concerning suicide prevention programs implemented in each region 3 years prior to the study will be recorded by each regions.

#### Intervention program process monitoring

Every 6 months, each regional leader will collect information regarding the implemented projects described in the intervention program manual.

#### Data collection during the study

##### (1) Information on suicides

After consent is obtained for the use of designated statistics for other purposes, information regarding the number of suicides in the participating regions will be collected. Death certificates from the Vital Statistics records from 2006, 2007, 2008, and 2009 for the intervention and control regions will be used to collect the following data items: International Classification of Diseases 10th Revision (ICD-10) code for intentional self-harm (ICD-10 codes X60–X84), residence of individuals who committed suicide (municipality codes), cause of death, external cause of death (ICD-10 code), measure of suicide (ICD-10), sex, age, reported place (municipality codes), and identification number.

##### (2) Information regarding suicidal behavior

Information regarding "self-harmed" individuals transported by ambulance will be collected from emergency reports.

The following information will be collected regarding "self-harmed" individuals every 6 months: type of transportation, date of notification, residence address, destination address, incidence location, severity (death, severe, moderate, mild, other), sex, age, and means of self-harm infliction.

##### (3) Demographic information

Total population numbers in the regions will be collected every year between 2006 and 2009.

##### (4) Information regarding ongoing suicide prevention programs

Information regarding the existence and implementation of suicide prevention programs in each participating region will be collected every 6 months.

#### Responsibility for data collection

Regional leaders are responsible for collecting data from each municipality and sending the data set to the data management center in a timely manner.

#### Data management

Collected data will be exclusively managed by the data management center. The data set will comply with the data management procedures and the Personal Information Protection Law. The data set will be periodically duplicated and saved as a backup file.

### Outcomes

#### Primary outcome

The incidence of suicidal behavior (completed suicides and suicide attempts excluding mild cases reported on emergency reports).

#### Secondary outcomes

(1) Incidence of completed suicides.

(2) Incidence of suicide attempts.

### Statistical analysis

#### Primary analysis

In the primary analysis, the incidence of suicidal behavior will be calculated based on the number of suicidal behavior per person-year for the annual population. Data obtained will include the incidence of suicidal behavior and its 95% confidence intervals adjusted by sex, 5-year age group, and regional characteristics. This data will be compared between the intervention and control regions in "Group 1".

The significance level will be set at 0.05 for the two-sided test, and will be adjusted in the final analysis based on the methods of O'Brien and Fleming [12] for interim analysis.

Additionally, regression analysis will also be performed to examine the interactions among sex, 5-year age group, and regional characteristics. A statistician in the study group will determine the analysis plan, whereas a different independent statistician will perform the interim analysis. The independent statistician will not contribute to the revision of the statistical analysis plan after interim analysis.

#### Interim analysis and rules for stopping or revising the study protocol

The interim analysis in "Group 1" will be performed 2 years after the study's implementation to evaluate the achievement of the primary objectives. Multiplicity will be adjusted using the methods of O'Brien and Fleming, in order to maintain Type-1 error at 0.05 for the two-sided test. The results will be reported to the Central Research Ethics Committee, which is expected to make recommendations to the J-MISP director to either stop the study or revise the study protocol if the primary objective of the study has already been achieved or is unlikely to be achieved.

#### Secondary analysis

In addition to the primary analysis, it will also be evaluated whether the primary outcome (the incidence of suicidal behavior) is also significantly reduced in intervention regions of "Group 2" areas, as a consequence of implementation of the program, when compared to control regions. The incidence of suicidal behavior will be investigated in Groups 1 and 2 combined. The analysis will be performed using the primary analysis plan described above.

Secondary outcomes will also be examined in order to determine whether the rate of completed suicides and suicide attempts – including individuals with severe, moderate, and mild self-harm transported to a hospital – is significantly reduced in the intervention regions, when compared to the control regions in "Group 1" and "Group 2". The same will be examined for both groups combined.

Subgroup analysis of the primary and secondary outcomes will be performed by sex, 5-year age group, and regional characteristics in "Group 1", "Group 2", and both groups combined. In addition, the incidence of suicidal behavior adjusted by sex and 5-year age group in the intervention and control regions will be calculated using the model population in 1985 as a reference population. Because of the exploratory nature of the secondary analysis, no adjustment for multiplicity will be made.

### Ethical considerations

The rights and welfare of the participating residents will be protected according to the World Medical Association Declaration of Helsinki Ethical Principles for Medical Research Involving Human Subjects. The study will comply with the ethical guidelines of the Ministry of Education, Culture, Sports, Science and Technology, as well as the Ministry of Health, Labour, and Welfare. Ethical validity, including safety, scientific legitimacy, and the reliability of results are to be ensured. This study will also comply with the ethical guidelines for epidemiologic studies and the Personal Information Protection law. The NOCOMIT-J principal investigator and the J-MISP director will be responsible for the protection of personal information during the study.

The data collected in this study will not include personal identification that would enable individuals to be identified. The data management center will collect only anonymous data.

### Stopping of the study

The J-MISP director is to inform the principal investigator of the NOCOMIT-J of the decisions of the Central Research Ethics Committee in the cases described below to discuss whether the study in each region or all of them should be discontinued.

a) The results of the interim analysis do not satisfy the standards set by the committee.

b) Safety issues that might affect the conduct of future studies arise from the results of interim analysis or the results of periodic monitoring.

c) The Local Research Ethics Committee of a region retracts consent to participate.

### Revision of the study protocol and due process

The J-MISP director is to inform the NOCOMIT-J principal investigator of the decisions of the Central Research Ethics Committee as soon as possible, when the Central Research Ethics Committee recommends that the study be redesigned due to the emergence of safety issues based on the interim analysis, periodic monitoring, and/or emergence of serious issues that might affect the conduct of future studies. The J-MISP director is to call a meeting of the study group and discuss the revision of the study protocol. If a recommendation to revise the study protocol is made, the principal investigator of the NOCOMIT-J will propose the revised study protocol as soon as possible and submit the proposal to the J-MISP director.

The J-MISP director will deliberate and approve the proposal at the Central Research Ethics Committee meeting and adopt the revision of the study protocol after deliberation in the steering committee. The study group management office will inform all of the participating researchers, and regional leaders will submit the proposed revision to the Local Research Ethics Committee and local government in each of the participating regions. The revision of the study protocol is to be implemented when approved.

### Study monitoring

#### Periodic monitoring

The regional leaders will periodically (once every 6 months) submit reports evaluating the progress of the study to the intervention program committee. The intervention program committee will submit a process evaluation monitoring report to the study group management office and J-MISP administration office once every 6 months. The J-MISP administration office will consider the progress of the research and submit the process evaluation monitoring report to the progress control committee and the Central Research Ethics Committee of the J-MISP.

The data management center will submit an event monitoring report to the J-MISP administration office. The office will submit event monitoring reports to the progress control committee, Central Research Ethics Committee, and the study group management office. The event monitoring report, which will contain the results of the analysis separated by intervention and control groups, will be submitted to the progress control committee and Central Research Ethics Committee. The results of the data analyzed from both groups combined will be submitted to the study group management office.

The progress control committee will examine the monitoring reports and submit the evaluation to the J-MISP director. The Central Research Ethics Committee will examine the monitoring reports as a third party, and make recommendations to revise the study protocol or discontinue the study to the J-MISP director when and if ethical problems, such as safety and efficacy issues, arise.

#### Monitoring reports

The process evaluation monitoring report will contain the following:

(1) An evaluation of the implementation progress of the study.

(2) A program process evaluation.

(3) Reports of individual cases and events requiring intervention and other information.

The event monitoring report will contain the following:

(1) Data on the incidence of suicidal behavior (total number of both suicide completions and attempts) in the intervention and control groups, etc.

(2) Other relevant information.

## Discussion

The study presented here is designed to evaluate the effectiveness of the community-based suicide prevention program in seven participating areas. Because treatment and prevention of suicide are complex and encompass many factors, success

will need multi-sector collaboration. We hope that the results of NOCOMIT-J will help to develop effective strategies to reduce future suicide rate in Japan.

## Competing interests

The authors declare that they have no competing interests.

## Authors' contributions

All authors participated in the design of the study. All authors contributed to the writing of the manuscript and have approved the final manuscript.

## Pre-publication history

The pre-publication history for this paper can be accessed here:


